# Supplementary material for: The functional organization of chromosome territories in single nuclei during zygotic genome activation
Source: bioRxiv. 2025 Apr 6:2025.04.06.647428. Preprint. [Version 1] doi: 10.1101/2025.04.06.647428 (PMC12456445; doi:10.1101/2025.04.06.647428)
Supplement: Supplement 5 [file NIHPP2025.04.06.647428v1-supplement-5.pdf]

bioRxiv preprint doi: https://doi.org/10.1101/2025.04.06.647428; this version posted April 6, 2025. The copyright holder for this preprint (which was not certified by peer review) is the author/funder, who has granted bioRxiv a license to display the preprint in perpetuity. It is made available under aCC-BY 4.0 International license.

Supplemental information

The functional organization of chromosome territories in single nuclei during zygotic genome activation

Akshada Shankar Ganesh, Taylor M. Orban, Romir Raj, Peter I. Fatzinger, Anna Johnson, Sean M. Riccard, Akhmed Zhanaidarov, Mayu Inaba, and Jelena Erceg

bioRxiv preprint doi: https://doi.org/10.1101/2025.04.06.647428; this version posted April 6, 2025. The copyright holder for this preprint (which was not certified by peer review) is the author/funder, who has granted bioRxiv a license to display the preprint in perpetuity. It is made available under aCC-BY 4.0 International license.

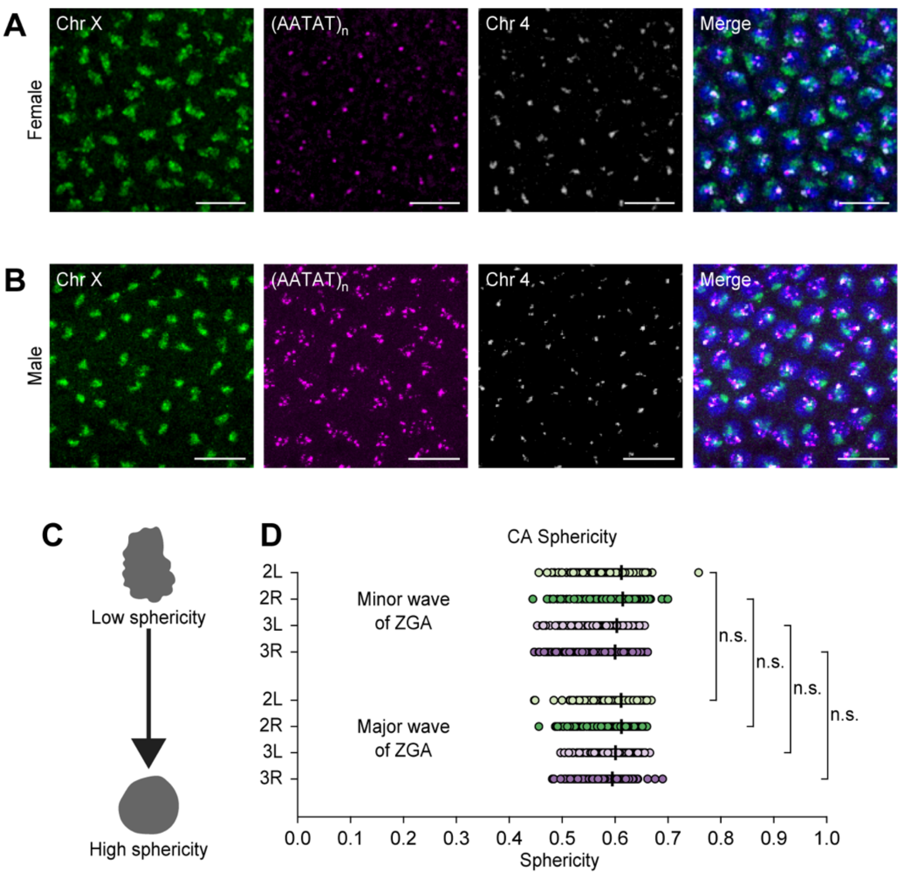

Figure S1. Chromosome X in female and male embryos and sphericity of CAs.

(A and B) Chromosome X (green), satellite repeat probe $(AATAT)_n$ (magenta), and chromosome 4 (gray) in female (A) and male (B) embryos during major wave of ZGA. Total DNA by Hoechst stain (blue). Bar = 10 µm. (C) Illustration showing low and high sphericity. (D) CA sphericity between the minor and major waves of ZGA. Median, solid line; at least three replicates; $n \geq 300$ nuclei; n.s., not significant, Mann-Whitney two-sided *U* test.

bioRxiv preprint doi: https://doi.org/10.1101/2025.04.06.647428; this version posted April 6, 2025. The copyright holder for this preprint (which was not certified by peer review) is the author/funder, who has granted bioRxiv a license to display the preprint in perpetuity. It is made available under aCC-BY 4.0 International license.

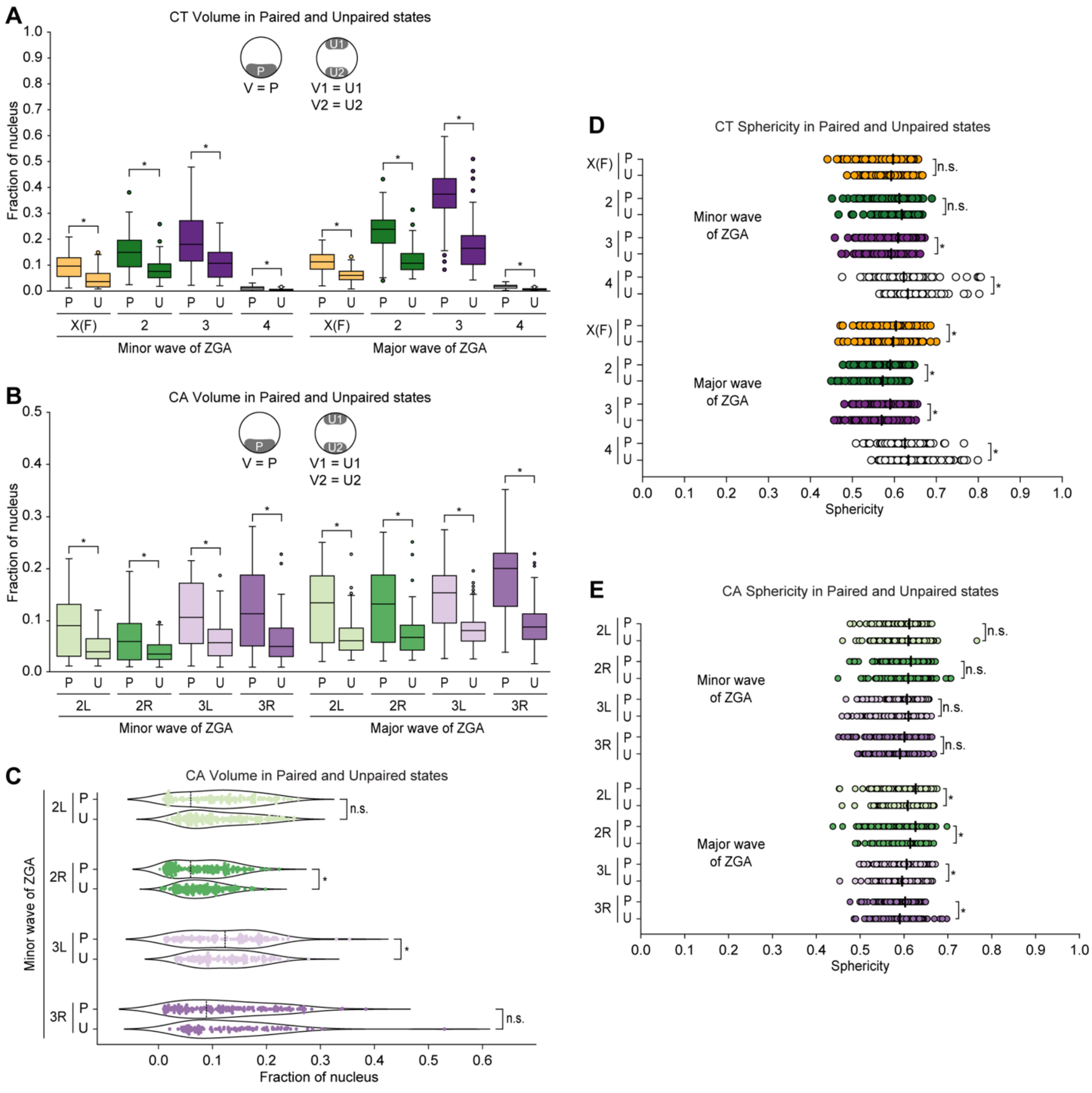

Figure S2. Volume and sphericity changes of CTs and CAs based on pairing.

(A) Normalized CT volume changes between paired homologs to individual unpaired homologs from the minor to major waves of ZGA. X(F), chromosome X in females; P, paired; U, unpaired; U1, unpaired homolog 1; U2, unpaired homolog 2; V, volume; at least three replicates; n ≥ 128 nuclei; *p ≤ 1.26×10^{-9}, Mann-Whitney two-sided *U* test. (B) Normalized volume differences in arms of paired homologs to arms of individual unpaired homologs during

bioRxiv preprint doi: https://doi.org/10.1101/2025.04.06.647428; this version posted April 6, 2025. The copyright holder for this preprint (which was not certified by peer review) is the author/funder, who has granted bioRxiv a license to display the preprint in perpetuity. It is made available under aCC-BY 4.0 International license.

ZGA. P, paired; U, unpaired; U1, unpaired homolog 1; U2, unpaired homolog 2; V, volume; at least three replicates; n ≥ 109 nuclei; *$p \leq 2.67\times10^{-4}$, Mann-Whitney two-sided *U* test. (C) Normalized CA volume differences in arms of paired homologs to the combined volume of two unpaired homologs during ZGA. P, paired; U, unpaired; local minima, dashed line; at least three replicates; n ≥ 103 nuclei; *$p \leq 1.12\times10^{-3}$, n.s., not significant, Levene's test. (D) CT sphericity changes between paired and unpaired homologs during ZGA. X(F), chromosome X in females; P, paired; U, unpaired; median, solid line; at least three replicates; n ≥ 148 nuclei; *$p \leq 3.58\times10^{-2}$, n.s., not significant, Mann-Whitney two-sided *U* test. (E) CA sphericity of paired and unpaired homologs during ZGA. P, paired; U, unpaired; median, solid line; at least three replicates; n ≥ 150 nuclei; *$p \leq 3.77\times10^{-4}$, n.s., not significant, Mann-Whitney two-sided *U* test.

bioRxiv preprint doi: https://doi.org/10.1101/2025.04.06.647428; this version posted April 6, 2025. The copyright holder for this preprint (which was not certified by peer review) is the author/funder, who has granted bioRxiv a license to display the preprint in perpetuity. It is made available under aCC-BY 4.0 International license.

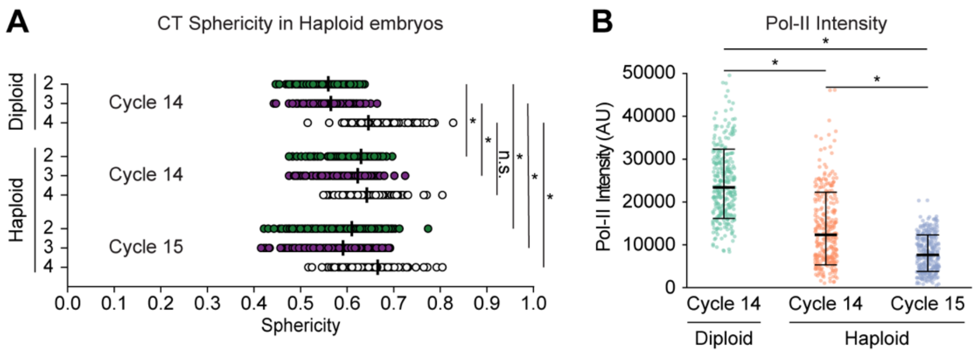

Figure S3. Sphericity of CTs and RNA Pol II intensity for diploid and haploid embryos.

(A) CT sphericity in diploid and haploid embryos during major wave of ZGA. In diploid embryos, only individual unpaired homolog sphericity was used. Median, solid line; at least three replicates; n ≥ 300 nuclei; *p ≤ 1.87×10^{-7}, n.s., not significant, Mann-Whitney two-sided *U* test. (B) Distribution of RNA Pol II fluorescence intensity (a.u.) within nucleus for diploid and haploid embryos during major wave of ZGA. At least three replicates; n ≥ 300 nuclei; *p ≤ 3.33×10^{-18}, Mann-Whitney two-sided *U* test.

bioRxiv preprint doi: https://doi.org/10.1101/2025.04.06.647428; this version posted April 6, 2025. The copyright holder for this preprint (which was not certified by peer review) is the author/funder, who has granted bioRxiv a license to display the preprint in perpetuity. It is made available under aCC-BY 4.0 International license.

Table S1. Oligopaint summary and CT/CA changes during minor and major waves of ZGA. *See separate excel sheet.*

(A) Summary of Oligopaint probes designed. (B) Primers for Oligopaint probes. (C) Normalized CT volume and (D) sphericity during ZGA. (E) Normalized CA volume and (F) sphericity during ZGA. X(M), chromosome X in males; X(F), chromosome X in females.

Table S2. Pairing of CTs and CAs during ZGA. *See separate excel sheet.*

(A) CT pairing and (B) CA pairing during minor and major waves of ZGA. (C) Normalized CT volume and (D) CA volume changes between paired homologs to the combined volume of two unpaired homologs. (E) Normalized CT volume and (F) CA volume differences between paired and individual unpaired homologs. (G) CT sphericity and (H) CA sphericity between paired and unpaired homologs. P, paired; U, unpaired.

Table S3. CT and RNA Pol II dynamics in haploid embryos. *See separate excel sheet.*

(A) Nuclear volume, (B) normalized CT volume, and (C) HLB volume in diploid and haploid embryos during major wave of ZGA. (B and C) In diploid embryos, only individual unpaired homolog volumes were used. (D) CT sphericity differences in diploid and haploid embryos. In diploid embryos, only individual unpaired homolog sphericity was used. (E) RNA Pol II fluorescence intensity (a.u.) within nucleus for diploid and haploid embryos.

bioRxiv preprint doi: https://doi.org/10.1101/2025.04.06.647428; this version posted April 6, 2025. The copyright holder for this preprint (which was not certified by peer review) is the author/funder, who has granted bioRxiv a license to display the preprint in perpetuity. It is made available under aCC-BY 4.0 International license.

Table S4. CT and nuclear volume measurements in transcription inhibited embryos. *See separate excel sheet*.

(A) Nuclear volume, (B) normalized CT volume, and (C) CT pairing in transcription inhibited embryos during major wave of ZGA.
